# Applications of the scoliosis width-to-length ratio for guiding selection of the surgical approaches of degenerative lumbar scoliosis

**DOI:** 10.1186/s12891-016-0904-3

**Published:** 2016-02-01

**Authors:** Chuan-jie Jiang, Yong-jun Yang, Ji-ping Zhou, Shu-qiang Yao, Kai Yang, Rui Wu, Yuan-chao Tan

**Affiliations:** Department of Orthopaedics, Shandong Wendeng Orthopedic and Traumatic Hospital, No.1 Fengshan Road, Wendeng, Shandong 26400 China

**Keywords:** Degenerative lumbar scoliosis, Spinal stenosis, Short fusion, Long fusion

## Abstract

**Background:**

There does not exist a comprehensive parameter for guiding selection of short or long segment fusion for degenerative lumbar scoliosis (DLS). The aim of our study was to investigate the applications of the width-to-length ratio in guiding selection of the surgical approaches for DLS.

**Methods:**

A retrospective analysis was performed of 142 patients with DLS who underwent operative treatments from July 2000 to January 2012. The scoliosis width-to-length ratios were measured and used as a grouping criterion of surgical approaches. The Oswestry disability index (ODI) was used to evaluate the clinical outcomes. Radiological parameters such as Cobb’s angle of main curve, Cobb’s angle of compensatory curve were all measured.

**Results:**

For patients with width-to-length ratio less than 0.36, the short segment group had better short-term postoperative outcomes with regard to Cobb’s angle of main curve, Cobb’s angle of compensatory curve and ODI scores compared to the long segment group. However, for patients with width-to-length ratio greater than 0.36, the postoperative outcomes for the long segment group were better compared to the short segment group.

**Conclusions:**

The scoliosis width-to-length ratio can provide a comprehensive preoperative assessment of the severity of the DLS and guiding selection of a therapeutic treatment regimen. Further studies with a larger number of samples and longer term of follow up are warranted.

## Background

Degenerative lumbar scoliosis (DLS) is one of the commonly encountered diseases in the older adult patients with spinal pain [1]. DLS was defined as coronal curvature (major lumbar curve) of ≥10° measured by the Cobb method [2] with the apex between L2 and L4. It is often associated with a combination of neurogenic claudication, radicular pain, and symptoms of lower back pain [3]. Radiological examination often exhibited various forms of instability, such as degenerative spondylolisthesis, lateral listhesis, the collapse of the intervertebral space, lumbar lordosis change, and pelvic tilt. Conservative treatment is often recommended for patients who have no significant stenotic, radicular, and/or back pain symptoms, including curves < 30° with < 2 mm of subluxation with anterior osteophytes [4]. Surgical interventions are required for patients who failed to respond to the conservative treatment [5]. Surgical treatments mainly include decompression and fusion or decompression alone [6–8]. Currently, there has been no consensus regarding the best surgical approaches for patients with DLS [9, 10]. Inappropriate surgical treatments may produces substantial postoperative complications [11]. In addition to the patients’ symptoms and signs, preoperative radiological evaluation is essential for the selection of therapeutic regimens of DLS. Currently, there have been many radiological parameters regarding DLS, such as Cobb angle, global coronal alignment (trunk shift-defined as the horizontal distance between the midpoint of the seventh cervical vertebra and the center of the pelvis on coronal plane), lumbar lordosis index, pelvic incidence (PI), pelvic rotation and pelvic tilt (PT), sacrum slop (SS), lumbo-pelvic index, sagittal vertical axis (SVA), thoracic kyphosis (TK). However, due to those parameters are relatively independent and of different degree of importance, the above parameters cannot be applied in combination to conduct a comprehensive evaluation of scoliosis. To our knowledgement, there does not exist a comprehensive parameter for guiding selection of short or long segment fusion or decompression alone for DLS [12–14]. The lack of a reliable parameter as a basis of the selection of the surgical is likely to cause inappropriate selection of the surgical regimens and obviously different surgical regimens among different physicians and correspondingly lead to unsatisfying postoperative outcomes. Therefore, we propose a new parameter-the scoliosis width-to-length ratio and apply it in the evaluation of the severity of the scoliosis. The parameter can make a three-dimensional comprehensive evaluation of the DLS quantitatively and provide a guidance of selection of a therapeutic treatment regimen which can reduce or avoid the risk of progression of the disease or incomplete symptomatic relief after surgery. The aim of our study was to investigate the applications of the width-to-length ratio in guiding selection of the surgical approaches for DLS.

## Methods

### Patients

This study was approved by the institutional review board of the Shandong Wendeng orthopedic and trauma hospital and conducted in accordance with the Declaration of Helsinki. Written informed consent was obtained from each subject.

We retrospectively analyzed the clinical data of patients with DLS treated with decompression combined with pedicle screw fixation and fusion by 3–4 spine surgeons at one institution between July 2000 and January 2012. Inclusion criteria were shown as follows: (1) patients who had a Cobb angle ≥ 10° before surgery; (2) patients older than 50 years; and (3) patients with a minimum 24-month follow-up. Exclusion criteria were shown as follows: (1) patients younger than 50 years; (2) patients with a history of previous spine surgery, trauma and infection, adolescent scoliosis or kyphosis, ankylosing spondylitis or osteoporotic vertebral fracture, and metabolic spinal pathology; and (3) patients with a preoperative Cobb angle < 10° [15].

A total of 142 patients were included in this study. Physical examinations showed 63 patients with the hypesthesia, decreased muscle strength and 22 presented with the saddle hypesthesia. Of these 63 patients, 45 cases with changes involved only one nerve segment, 15 cases involved two nerve segments, and the remaining 3 cases involved more than two nerve segments. The radiographic and MRI examinations showed that most of the patients had lumbar scoliosis and different levels of spinal stenosis to some extent, with 115 patients showing one compensatory curve, 22 patients showing two compensatory curves, 5 patients showing no compensatory curves [16]. All of the patients received conservative treatment for at least three months before they were admitted to our hospital. During the period of conservative treatment, their symptoms did not improve dramatically or got even worse.

### Radiographic assessment

**Fig. 1:**
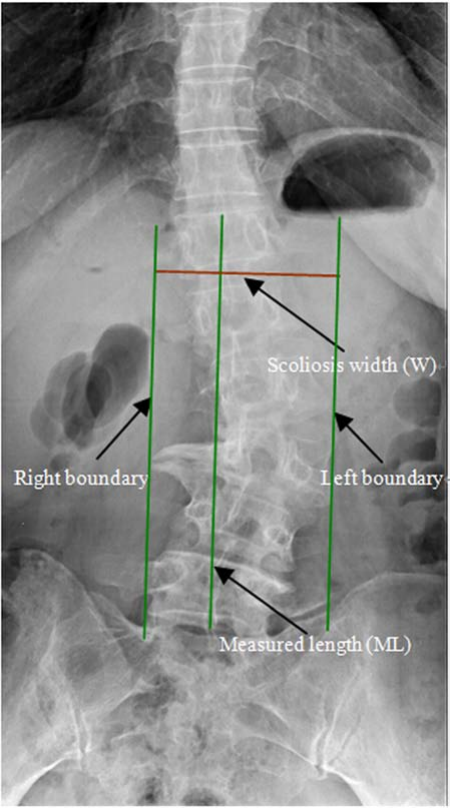
The scoliosis width (W) was defined as the linear displacement distance between the boundary lines of the scoliosis

**Fig. 2:**
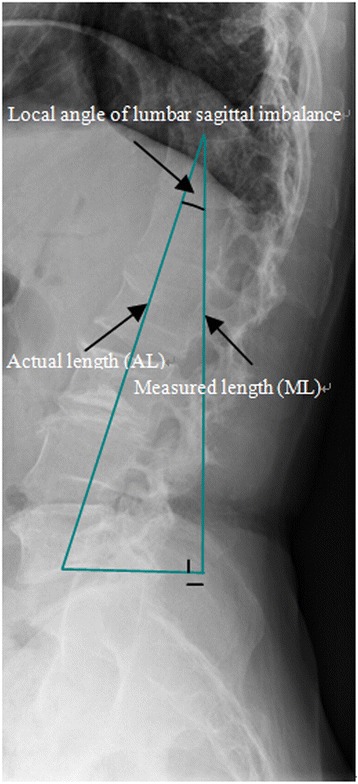
The scoliosis actual length (AL) was calculated by the cosine of the angle between the measured length (ML) and the actual length (AL)

Anteroposterior and lateral radiographs were reviewed preoperatively and at the final follow up periods. Standard X-ray radiographs in the standing position were taken with an unified distance value of 120 cm between the negative film and the bulb tube. The anteroposterior film in a standing position was taken with patients facing bulb tube. Right lateral X-ray films in a standing position were taken with centering at L3 level. The following parameters including the Cobb’s angle of the main curve, the Cobb’s angle of the compensatory or secondary curve, Bending Cobb’s angle, top vertebra, neutral vertebra, thoracolumbar kyphosis angle, lumbar lordosis, subluxation or lateral listhesis, and coronal and sagittal imbalance parameters were measured. We measured and calculated lumbar scoliosis width-to-length ratio using the following formation: W/AL = scoliosis width (W)/scoliosis actual length (AL). Here the scoliosis width (W) was the total width of the lumbar scoliosis segment (not including osteophytes). The specific method to calculate W is: on the anteroposterior X-ray films, we first connected the midpoints of the T12 upper endplate and the L5 lower endplate, the measured length (ML) was the distance between the midpoints of the T12 upper endplate and L5 lower endplate. Then extended this line parallelly to both sides, until it intersected with the two boundary lines of the scoliosis. Linear displacement distance between the two lines was defined as the scoliosis width (W) (Fig. 1). The scoliosis actual length (AL) was calculated as the results of the measured length (ML) divided by the cosine of the local angle of lumbar sagittal imbalance on the lateral X-ray films (Fig. 2). The local angle of lumbar sagittal imbalance was calculated using the following method: on the lateral X-ray films, first draw a straight line between the midpoints of T12 upper endplate and L5 lower endplate, the local angle of lumbar sagittal imbalance was then defined as the angle between this straight line and the perpendicular line through the midpoint of T12 upper endplate. Finally, lumbar scoliosis width-to-length ratio (W/AL) was used to quantify the severity of the scoliosis (coronal section). The reason that we cannot measure the AL on the anteroposterior film directly is due to the directly measured AL is virtually the projection length instead of the actual scoliosis length. The directly measured AL does not consider the severity of the antero-posterior imbalance and thus is not correct. In order to eliminate the effects, we introduce the trigonometric function. The goal of measuring ML on the anteroposterior films is to simultaneously measuring the scoliosis W and ML with an identical plotting scale which can ensure the two parameters more comparable. To reduce any observation bias, two independent investigators repetitively performed all radiographic measurements. The intra- and interobserver reliabilities of all measurements were assessed using the kappa value. In this study, the intra-observer kappa value of the measurements of W/AL ratio was 0.84 (range, 0.82–0.94) and the inter-observer kappa value of the measurements of W/AL ratio was 0.86 (range, 0.82–0.96), suggesting substantial perfect agreement.

### Grouping

According to the definition of the width-to-length ratio, the magnitude of the W/AL ratio can reflect the severity of the scoliosis. We calculated the W/AL ratios of the included 142 cases and achieved a mean value of 0.3622 ± 0.0585. Hence a threshold value of 0.36 was selected for grouping. According to the W/AL ratio as well as other parameters such as degree of spondylolisthesis, degree of spinal stenosis, Cobb’s angle of the main curve, the Cobbs’s angle of the compensatory or secondary curve, lumbar lordosis angle, subluxation or lateral listhesis, and coronal and sagittal imbalance parameters, the 142 patients were divided into four groups followed by different surgical treatments: Group A: 59 patients with lumbar scoliosis width-to-length ratio (W/AL) < 0.36 were treated with short segment fusion; Group B: 28 patients with lumbar scoliosis width-to-length ratio (W/AL) > 0.36 were treated with short segment fusion due to their ages and physique reasons; Group C: 21 patients with lumbar scoliosis width-to-length ratio (W/AL) < 0.36 were treated with long segment fusion due to the presence of multi segmental lumbar disc herniation or unstable conditions; Group D, 34 patients with lumbar scoliosis width-to-length ratio (W/AL) > 0.36 were treated with long segment fusion. It should be noted that short segment fusion in Group A and B were performed only on the segments responsible for deformity, while long segment fusion in Group C and D were performed on all deformities within the deformity and beyond.

### Surgical techniques

The surgical segments were determined by a senior surgeon depending on the patients’ symptoms, physique, signs and imaging examination results. For patients with mild scoliosis, the surgeon used mainly short segment fusion, focused on the responsible and unstable or initiated segment of spinal stenosis and scoliosis, which were typically less than or equal to three segments. For patients the severe scoliosis or even obvious coronal imbalance, the surgeon fused most segments within or beyond the deformity (long segment fusion). For patients with small scoliosis but with multisegment degeneration or spinal canal stenosis, long segment fusion was performed in order to reduce the risk of disease progression and the adjacent segment disease. For patients who had severe scoliosis or even imbalance but with poor physical conditions or severe osteoporosis such as older patients, only short segment fusion was performed after consulting with their family. Parts of the small facets were also removed in some cases to get a better correction during surgery. Four to six weeks of bed rest after surgery were advised to walk with protective brace.

### Clinical evaluation

Clinical outcomes were assessed with the Oswestry disability index (ODI) [17].

### Statistical analysis

The clinical measurements and data were analyzed using SPSS 13.0 statistical software. Data were expressed as the mean and standard deviations. Comparisons of the clinical parameter of patients with DLS before surgery and the last follow-up period were performed by using paired t tests. Comparisons of the clinical parameters of patients with DLS between different groups were performed by analysis of variance. A P value of less than 0.05 was considered as statistically significant.

## Results

### Clinical parameters in the four groups

A total of 142 patients with DLS treated combined with pedicle screw fixation and fusion were included in this study. There were 33males and 109 females with an average age of 64.3 ± 8.4 years old (ranging from 54–78). Of these 142 patients, 69 patients had low back pain after movements, 70 had various degree of intermittent claudication and radicular pain. Table 1 shows the clinical characteristics of patients with DLS in the four groups.

**Table 1:** Patients’ baseline characteristics of the four groups

	Group A (*n* = 59)	Group B (*n* = 28)	Group C (*n* = 21)	Group D (*n* = 34)	*P* values
Age, years	62.9 ± 9.99	67.3 ± 8.54	58.2 ± 6.32	64.0 ± 3.29	0.383
Blood loss, ml	448 ± 121	612 ± 85	1118 ± 300	1250 ± 258	<0.001
Operation time, min	148 ± 30	170 ± 18	230 ± 61	236 ± 44	<0.001
Segment level of fusion	2.0 ± 0.87	2.5 ± 0.57	4.5 ± 1.1	4.6 ± 0.81	<0.001
No. of co-morbidities	0.97 ± 0.51	1.25 ± 0.5	1.11 ± 0.33	1.4 ± 0.55	0.32
No. of decompression	1.68 ± 0.78	2.0 ± 1.15	2.11 ± 0.93	2.33 ± 0.51	0.36
Hospital stay, days	16.45 ± 2.14	18.65 ± 1.56	15.35 ± 2.65	17.25 ± 1.54	0.006

Group A, patients whose W/L <0.36 and treated with short fusion

Group B, patients whoseW/L > 0.36 and treated with short fusion

Group C, patients whose W/L <0.36 and treated with long fusion

Group D, patients whoseW/L > 0.36 and treated with long fusion

### Radiological parameters between the four groups

Table 2 shows the pre-and post- radiological parameters between the four groups. The average Cobb’s angle of main curve in the group A, C, and D at the final follow-up was significantly lower than that of their preoperative values (*P* < 0.001). No significant improvement regarding the average Cobb’s angle of main curve was noted in the group B between postoperative and preoperative values (*P* > 0.05).

**Table 2:** The pre-and post- radiological parameters between the four groups

	Group A (*n* = 59)	Group B (*n* = 28)	Group C (*n* = 21)	Group D (*n* = 34)
Cobb’s angle of main curve				
Preoperative	13.59 ± 4.87	28.50 ± 17.60	13.44 ± 3.81	26.50 ± 4.68
Follow-up	5.87 ± 4.98	24.00 ± 14.91	4.22 ± 5.51	8.66 ± 4.50
P values	<0.001	0.09	<0.001	0.002
Cobb’s angle of compensatory curve				
Preoperative	5.31 ± 5.15	13.75 ± 6.55	2.56 ± 1.74	15.33 ± 1.63
Follow-up	1.68 ± 2.69	8.03 ± 2.57	1.05 ± 0.32	3.48 ± 2.28
*P* values	<0.001	0.16	0.005	<0.001
Scoliosis W/AL ratio				
Preoperative	0.34 ± 0.04	0.49 ± 0.09	0.35 ± 0.02	0.43 ± 0.03
Follow-up	0.29 ± 0.03	0.42 ± 0.03	0.30 ± 0.02	0.31 ± 0.03
*P* values	<0.001	0.19	<0.001	0.001

Group A, patients whose W/L <0.36 and treated with short fusion

Group B, patients whose W/L > 0.36 and treated with short fusion

Group C, patients whose W/L <0.36 and treated with long fusion

Group D, patients whose W/L > 0.36 and treated with long fusion

The average Cobb’s angle of compensatory curve in the group A, B, C, and D at the final follow-up was significantly lower compared to the preoperative values (*P* < 0.05).

The average W/AL ratios in the group A, C, and D at the final follow-up was significantly lower as comparison to the preoperative values (*P* < 0.05). No significant improvement with regard to the average Cobb’s angle of compensatory curve was noted in the group B between postoperative and preoperative values (*P* > 0.05).

### Functional outcomes

After surgery, the patients were followed up for a mean of 28.2 months ranged from 24–64 months. The clinical outcomes were assessed using the ODI. The ODI scores in the group A, and D at final visit were statistically significant improved in comparison to that of the preoperative values. In contrast, there were no statistically significant differences for Group B, C between the postoperative and preoperative values (*P* > 0.05) (Table 3).

**Table 3:** Oswestry disability index (ODI)

	Group A	Group B	Group C	Group D
Preoperative	59.94 ± 7.62	65.89 ± 5.78	57.57 ± 4.40	65.54 ± 18.73
Follow-up	24.68 ± 14.80^a^	55.54 ± 5.77^c^	55.55 ± 16.66^b^	23.38 ± 12.10^a^
Comparisons	35.26 ± 7.18	10.35 ± 0.01	2.02 ± 12.26	42.16 ± 6.63
*P* values	<0.001	0.184	0.695	0.002

^a^, vs preoperative, *P* < 0.05; ^b^,vs group A, *P* < 0.05; ^c^, vs group D, *P* < 0.05

Group A, patients whose W/L <0.36 and treated with short fusion

Group B, patients whoseW/L > 0.36 and treated with short fusion

Group C, patients whose W/L <0.36 and treated with long fusion

Group D, patients whoseW/L > 0.36 and treated with long fusion

**Fig. 3:**
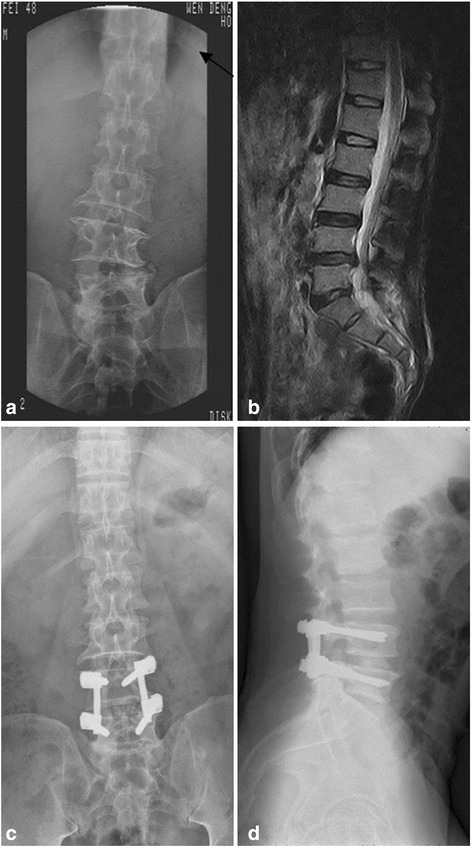
A 72 year old male patient with back pain and intermittent claudication for a year. a) preoperative radiograph showing degenerative scoliosis with 15° Cobb angle, L3/4 interval tip angle, 0.34 of W/AL. b) preoperative MRI showing L4/5 disc herniation, spinal stenosis, and degenerative L4/5 instability. c) postoperative anteroposterior radiography showing good fixation and fusion without secondary scoliosis. d) postoperative lateral radiography showing L4/5 segment fused and satisfying lumbar lordosis

**Fig. 4:**
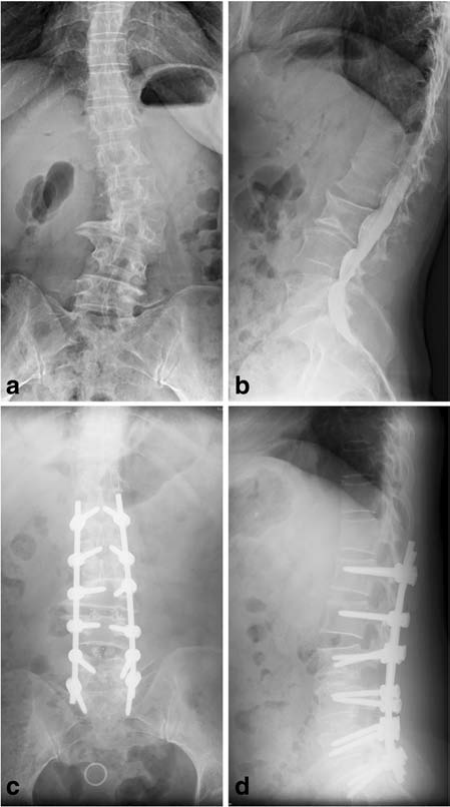
A 55 year old female patient with back pain and left leg numb pain for four months. a) preoperative radiograph showing degenerative scoliosis with 23°Cobb angle, L3/4 interval tip angle, 0.45 of W/AL. b) preoperative CT showing L4/5, L3/4, and L2/3 disc herniation. c) postoperative anteroposterior radiography showing good correction. d) postoperative lateral radiography showing satisfying lumbar lordosis

Further analysis of the ODI changes among different groups showed significant differences between Groups A and C (*P* < 0.05), suggesting that for patients with lumbar scoliosis width-to-length ratio lesser than 0.36, the short segment fusion treatment is more appropriate compared with the long segment fusion. Similarly, statistically significant differences was observed between Group B and Group D (*P* < 0.05), indicating that the long segment fusion may yield better clinical outcomes when the W/AL ratio was greater than 0.36. Figs. 3 and 4 show the preoperative and postoperative radiographs of two representative cases in this study.

## Discussion

Different surgical treatment approaches should be utilized due to the difference of the severity of the DLS. Decompression alone has been demonstrated to be invasive and associated with better postoperative outcomes in selected patients [18]. However, The procedure of decompression alone is usually not recommended because it can lead to further collapse and instability, especially at the apex of the degenerative curve [3, 19, 20]. Short segment fusion involves less surgical morbidity and represents an attempt to preserve lifestyle and activity levels that could be affected by a definitive fusion of the entire deformity. However, even with careful selection, a subset of patients who undergo limited procedures can later become symptomatic because of deformity progression, worsening sagittal or coronal imbalance, failure of the previous fusion, new instability, or adjacent segment degeneration [21–23]. With regards to the long segment fusion, it usually reaches or even crosses the boundaries of the deformity, involved with four or more segments [24]. However, it may cause massive lesion resulting in unsatisfying clinical outcomes [25]. Currently, there have been no consistent reference criteria for the selection of the aforementioned 3 surgical approaches. In this study, we introduced a new parameter of the scoliosis W/AL ratio to guide selection of the treatment of the scoliosis prior to the surgery, we achieved good postoperative outcomes. We set the mean value of the W/AL of the 142 patients with DLS (0.36) as the reference value. We found that the group A (short segment fusion group) and the group D (long segment fusion group) can improve significantly patients’ Cobb’s angle of main and compensatory curve and the average W/AL ratios. We also found that the group D had obvious increased operation time and the blood loss, which may increase the possibilities of the surgical risks. Attention should be paid to strengthen the preoperative evaluation for patients whose W/AL ratio less than 0.36.

Currently, there have been a variety of reference parameters for the evaluation of the DLS. However, these parameters alone can only partially evaluate the severity of the scoliosis and each parameter has its own importance in guiding selection of the surgical approach, which made it difficult to pinpoint a systematic evaluation indicator. Thus the comprehensive evaluation of the condition of the scoliosis will be depended on the surgeon’s experience, which may cause great difference in selection of the surgical approaches among surgeons and may eventually results in the significant difference concerning the surgical outcomes. In this study, the application of the scoliosis W/AL ratio can solve this problem. Horizontally, it evaluates the scoliosis curvature, vertebral lateral slip, subluxation, and utilizes the width (W) to indicate the severity of the lateral deformity. Vertically, it systematically evaluates the scoliosis, lumbar lordosis, dent or tilt of the intervertebral space. The length of the lumbar vertebra body will shorten with the progress of the severity of the scoliosis. Thus we utilize the length (L) to indicate its vertical deformity. In contrast to the uniqueness of other parameters, scoliosis W/AL ratio covers the scoliosis (including main curve and compensatory curve), kyphosis, intervertebral space tilt or dent, low or high LL or PT and other deformities, and thus can be used as a systemic evaluation parameter for the severity of the scoliosis. Meanwhile, the W/AL ratio avoids the influence of the radiographs and scale images from different periods at different hospitals. Furthermore, it quantifies the severity of the DLS, which significantly helps to the quantitative comparisons among them.

Our study showed that for patients with the W/AL ratio less than 0.36, relatively small scoliosis Cobb’s angle, mild vertebral lateral slip and subluxation symptoms, and satisfying spine balance, the short segment fusion [26] will produce an effective short-term outcomes. Our findings were consistent with a previous report Daubs et al. [18]. We found that short segment fusion can correct the main and compensatory curves of patients with scoliosis W/AL ratio of less than 0.36. Application of W/AL ratio can not only settle the responsible segments, but also significantly improve the Cobb angles. In addition, their postoperative symptoms tended to be improved, their spines were stable, and their main curve had no obvious deterioration. All these findings were consistent with a previous report by Seo et al. [26]. Our study suggests that it is not necessary to uniformly select the long segment fusion for all scoliosis patients, as it may cause excessive invasiveness to the spine and be associated with new secondary complications. Furthermore, excessive treatments will go against further treatments. Therefore, patients who had a scoliosis W/AL ratio of less than 0.36 and cannot tolerate the larger surgery due to poor physical condition, the short segment fusion on responsible segments can also be considered. Whereas for patients with W/AL ratio greater than 0.36, bigger Cobb’s angle, obvious vertebral lateral slip, subluxation, asymmetry of intervertebral space, dent, and smaller lumbar lordosis, the long segment fusion should be considered. This procedure can improve main and complementary curves and achieve obvious postoperative outcomes. While for patients with W/AL ratio around 0.36, the individualized treatment plan needs to be developed. Degeneration, instability, and sagittal balance of the adjacent segments of the involved segments should be considered to adjust the numbers of the segments pending process to avoid the progress of the scoliosis and secondary complications [27] or massive lesions. To sum up, the scoliosis W/AL ratio can objectively evaluate the severity of the scoliosis before surgery and contribute to the development of the optimal surgical strategies suited for the patients’ condition.

There are several limitations in this study. First, the follow-up time may be not long enough for short and long segment fusion although the patients were followed up for a mean of 28.2 months ranged from 24–64 months. A longer time of follow up will be warranted. Second, we did not measure and achieve the W/AL ratio of the non-scoliosis adults and thus we cannot compare it with the scoliosis W/AL ratio to explain the relationship between W/AL ratio and degeneration. Finally, we did not correlate the scoliosis W/AL ratio in specific spinal segment (e.g.T12-L2, L1-L5) with the patient’s symptoms and signs, especially in the apical vertebral regions while such information may be important for determining a surgical strategy.

## Conclusions

The scoliosis W/AL ratio can be used to conduct a preoperative evaluation of the severity of the DLS and guiding selection of a therapeutic treatment regimen. Further studies with a larger number of samples and longer term of follow up are warranted.

## Abbreviations

DLS: degenerative lumbar scoliosis
ML: measured length.
ODI: Oswestry disability index
PI: pelvic incidence
PT: pelvic tilt
SS: sacrum slope
SVA: sagittal vertical axis
SVA: sagittal vertical axis
TK: thoracic kyphosis
W/AL: width (W)/ actual length (AL)
